# Optical Enzyme Sensor for Urea Determination via Immobilized pH Indicator and Urease onto Transparent Membranes

**DOI:** 10.1100/tsw.2003.45

**Published:** 2003-07-01

**Authors:** Milka Krysteva, Mohamed Al Hallak

**Affiliations:** Department of Biotechnology, University of Chemical Technology and Metallurgy, Blvd. 8 Kl.Ohridski, 1756, Sofia, Bulgaria

**Keywords:** urea determination, optical enzyme sensor for urea, immobilized pH indicator, immobilized pH indicator and urease onto transparent membranes, urea determination for ecological purposes

## Abstract

Transparent triacetylcellulose membranes with immobilized pH indicator (neutral red) as well as with simultaneously immobilized urease and neutral red were used as optical sensors for determination of urea concentrations in model solutions. Decomposition of urea with the enzyme urease is accompanied by evolution of ammonia. This leads to the changes of the neutral red absorption, which is proportional to the substrate (urea) within certain concentration limits in model solution. As a result of the investigation, standard curves were plotted for determination of urea over the range of 1 to 500 mM using immobilized indicator and free urease. Simultaneous immobilization of indicator and urease permitted determination of urea in the interval 50 to 500 mM. The membrane used contained 0.169 U urease activity on an area of 1.7 cm. The standard curves were plotted using the linear region of the kinetic curves for the corresponding substrate concentrations. A possible scheme of the interaction between the activated triacetylcellulose membrane and the indicator and enzyme is proposed. The membranes obtained are suitable for repeated ecological applications where urea is to be determined.

## INTRODUCTION

Determination of urea in soil and water reservoirs for ecological purposes is of great practical importance. As is known, urease catalyzes urea decomposition. As a result, ammonia is evolved and the pH value increases. Ammonia may be determined by an ion selective electrode, an electrode for gaseous ammonia, and pH electrodes[1]. Enzyme electrodes with amperometric indication are also known[2]. Recently, neutral red was covalently immobilized on a triacetylcellulose membrane and used as pH sensor[3].

The present paper was aimed at transforming the membrane with immobilized indicator into an enzyme sensor in order to determine the concentration of urea by establishing the indicator absorption, which is changed by the ammonia, evolved during urea decomposition with urease.

## RESULTS AND DISCUSSION

### Determination of Urea with Immobilized Neutral Red and Free Urease

The spectral characteristics of immobilized neutral red indicator measured at pH values of 6.0 and 8.0, respectively, are shown in Fig. 1.

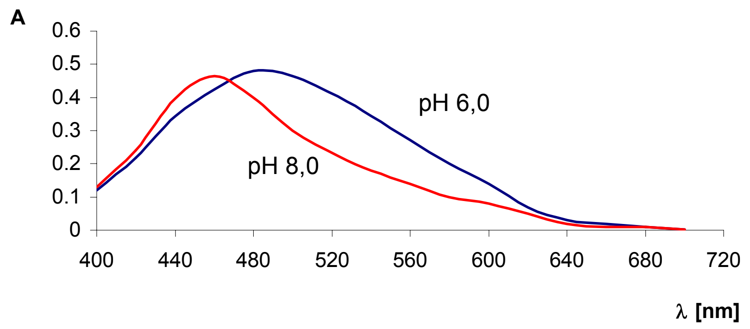

**FIGURE 1**. Spectra of neutral red after immobilization on a triacetylcellulose membrane.

The measurements were made at a wavelength of 520 nm where the alterations of the spectrum with changing pH are more distinct. Model urea solutions with concentrations ranging from 1 to 500 mM were used. The working volume (1.7 ml) of the cuvette had an enzyme activity of 2 U. Fig. 2 illustrates the kinetics of urea decomposition with free urease within the above interval.

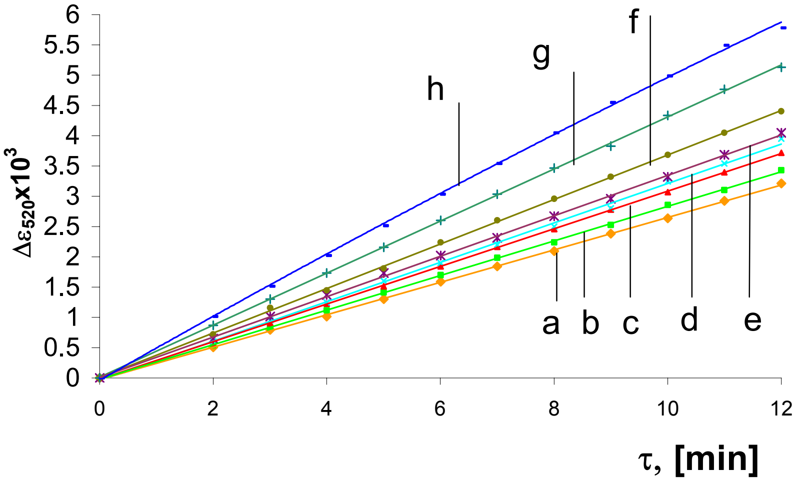

**FIGURE 2**. Kinetics of urea decomposition with free urease and immobilized indicator. Concentration in mM: (a) 2; (b) 5; (c) 10; (d) 30; (e) 50; (f) 100; (g) 300; (h) 500.

It is evident from the figure that at high concentrations (between 300 and 500 mM), saturation with substrate is observed after about 11 min. On the basis of the measurements made, a standard curve (Fig. 3) was plotted for determination of urea concentrations.

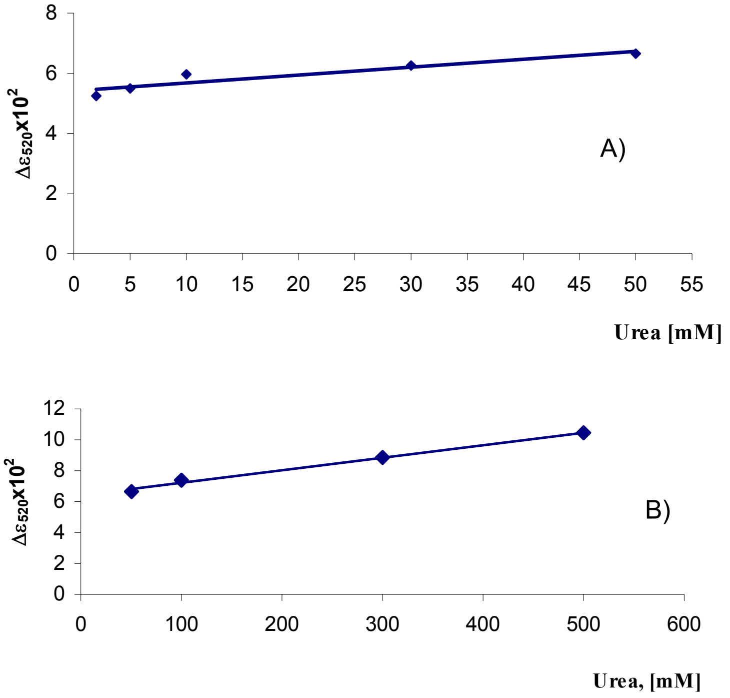

**FIGURE 3**. Standard curve for determination of the amount of urea using free enzyme and indicator immobilized on a triacetylcellulose membrane; in the range of 2 to 50 mM (3A); 50 to 500 mM (3B).

For the sake of convenience, this curve was divided into two parts, which permitted determination of low substrate concentrations in the range of 1 to 50 mM (Fig. 3A) as well as of higher concentrations (Fig. 3B) ranging from 50 to 500 mM. The data obtained using the standard curve were the result of 1-min measurements in the linear region of the curves in Fig. 2. These results allow one to assume that a wide range of urea concentrations can be measured using an indicator immobilized on a transparent membrane. The $K_m$ constant for free urease was found to be $1.2 \times 10^{-3}$ M.

## Determination of Urea with Urease and Neutral Red Immobilized Simultaneously onto a Transparent Triacetylcellulose Membrane

Determination of urea concentrations using immobilized enzyme and indicator was performed within the range 50 to 500 mM (Fig. 4). The studies were carried out with a membrane of 1.7 $m^2$. The activity of immobilized urease on it was 0.169 U and the optimum pH of immobilized enzyme was found to be 7.5, i.e., by 0.5 units higher than that of free enzyme (7.0). In contrast to the former case, urea decomposition occurred more slowly, which can be ascribed, on the one hand, to the lower enzyme activity introduced with the membrane and, on the other, to complications due to the proceeding of several heterogeneous reactions in the sequence: **penetration of the substrate [S] to the enzyme [E] → formation of the enzyme-substrate**

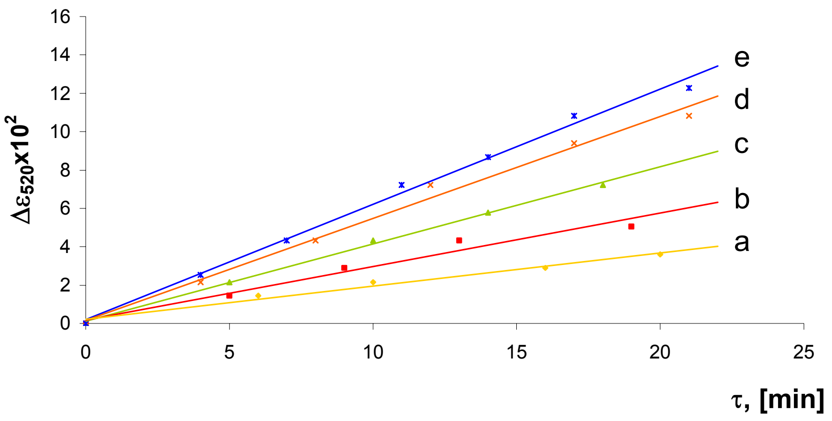

**FIGURE 4.** Kinetics of urea decomposition with immobilized urease and indicator on a triacetylcellulose membrane. Concentration in mM: (a) 100; (b) 200; (c) 300; (d) 400; (e) 500.

**complex [ES] → formation of the ammonia product [P] → pH change on the indicator [I] caused by the product, measured by the absorption [A].** Schematic representation of above-mentioned processes is presented in Fig. 5. At higher concentrations, saturation with substrate is observed after the 15th minute. On the basis of these studies, a standard curve was plotted (Fig. 6).

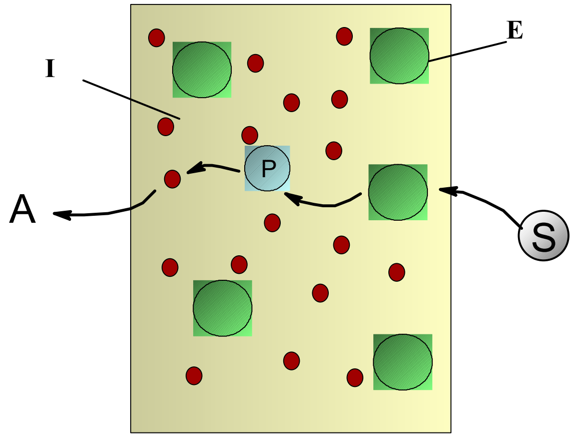

**FIGURE 5**. Schematic representation of the processes running on the membrane with immobilized indicator and enzyme when the substrate urea is added.

The data obtained were the result of 5-min measurements in the linear region of the curves in Fig. 4. The values obtained from the both curves are the result of at least three measurements. Since immobilization of neutral red occurred at pH 8.0, it could be assumed that probably an amino group of the dye was involved in its conjugation to the membrane. The immobilization of urease at pH 5.2 may be attributed to the Asn residues of the enzyme in agreement with the method used[4] (Fig. 7).

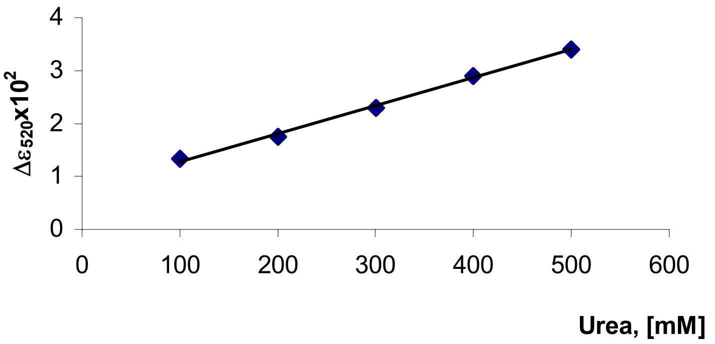

**FIGURE 6.** Standard curve for determination of the amount of urea using both enzyme and indicator immobilized on a triacetylcellulose membrane.

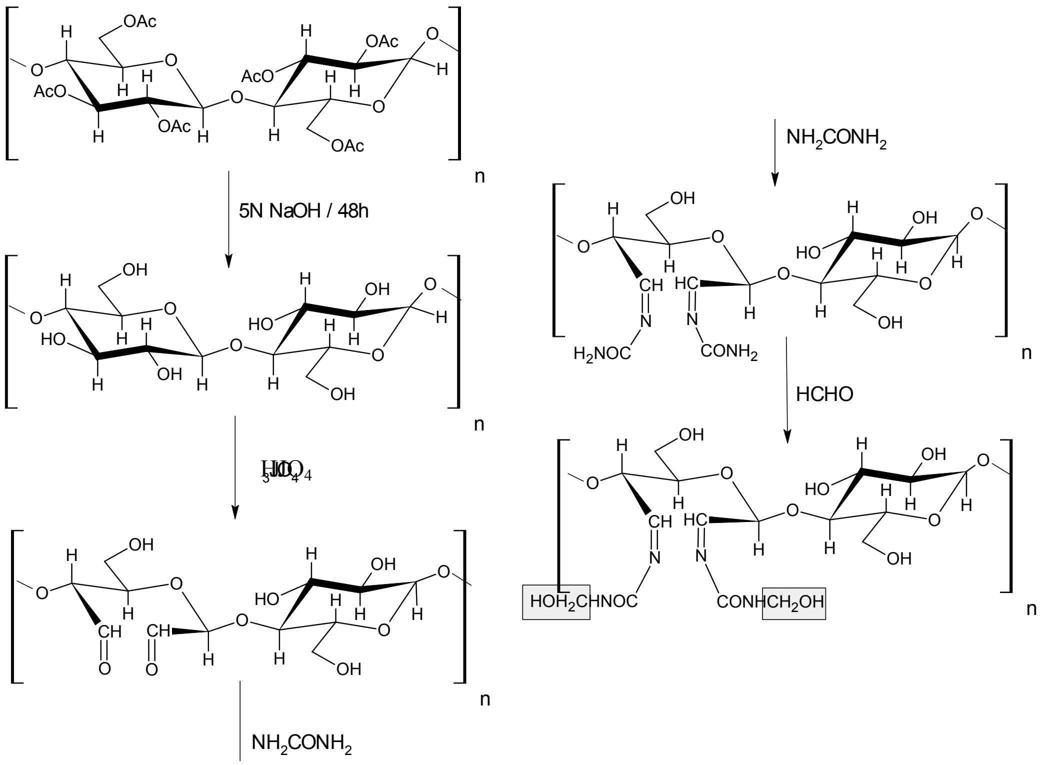

**(7A)**

**FIGURE 7**. Possible scheme of covalent binding of neutral red and urease to activated triacetylcellulose membrane; A – activation step; B – conjugation step.

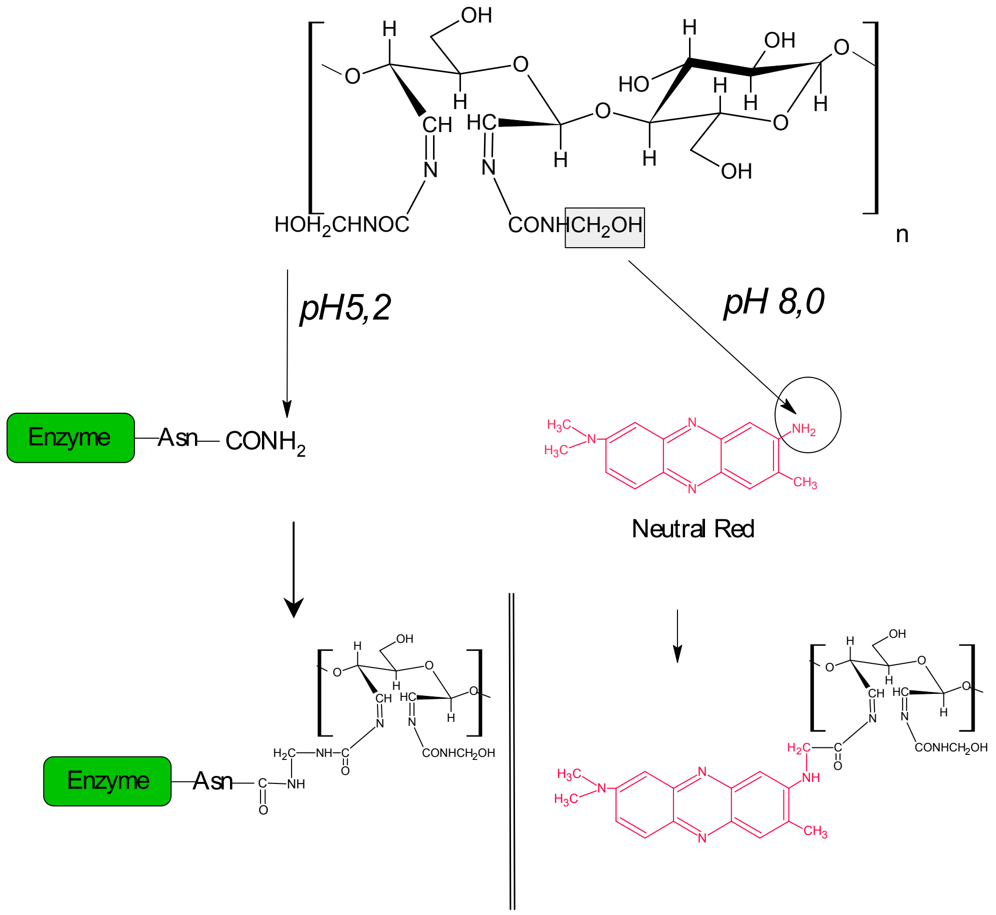

**FIGURE 7B**

On the other hand, the $K_m$ of $2 \times 10^{-2}$ M found for the immobilized urease indicates a decrease in the enzyme affinity to the substrate in comparison with free one. This finding maybe attributed to the relatively large indicator molecule, which hinders substrate penetration to the enzyme, the latter being, on its part, also immobilized to the matrix. The binding of the enzyme prior to the dye, i.e., at a lower pH, and not simultaneously with the indicator, preserves its higher activity. In view of the nonporous membrane surface one should not expect diffusion-dependent restrictions during the interaction of the enzyme with its substrate. Investigations on the stability of urease immobilized simultaneously with the indicator on the membrane have shown a drop of urease activity by about 20% in 60 days after permanent use. Membranes with immobilized indicator and urease should be stored wet at 4$^{o}$C.

## EXPERIMENTAL METHODS AND PROCEDURES

Neutral red and urease were supplied by Merck (Germany). Film membranes of triacetylcellulose were used as a matrix for indicator and enzyme immobilization after preliminary treatment as described[5].

## Activation of the Triacetylcellulose Membrane

The membrane (5 g) was treated with 5 N sodium hydroxide for 48 h with a view to its partial deacetylation, then washed with a large amount of distilled water in order to remove the hydroxide. The partly deacetylated membrane was treated with 0.25 M sodium periodate at 4°C in 0.1 M acetate buffer with pH 3.8 for 14 h in the dark. After that, the membrane was carefully washed with distilled water. The oxidized membrane was further treated with 15% urea for 14 to 16 h in the presence of 0.9% sulfuric acid at 60°C on a water bath with continuous shaking. The urea derivative formed on the cellulose membrane was stable. After careful washing with distilled water, the membrane can be stored at 4°C for an unlimited time.

## Activation of the Oxidized Membrane with Formaldehyde

The urea derivative on the membrane was treated with 12.5% formaldehyde at 45°C for 16 h in 0.1 M phosphate buffer (pH 7.5) on a water bath with continuous shaking. After repeated washing with distilled water the activated membrane was used again for the next treatment.

## Immobilization of Neutral Red onto the Activated Membrane

A 4-$cm^2$ membrane activated with formaldehyde was subjected to 24-h treatment with 10 ml 0.1 M phosphate buffer containing 20 mg neutral red at room temperature with continuous shaking. The treatment was performed at three different pH values: 7.0, 8.0, and 9.0. After that, the membrane with the immobilized indicator was washed carefully with distilled water and 3 N sodium chloride solution during continuous shaking until the complete removal of nonbonded indicator.

## Simultaneous Immobilization of Neutral Red and Urease onto the Activated Membrane

The membrane (4 $cm^2$) activated with formaldehyde and 20 mg urease was placed in 10 ml 0.1 M acetate buffer at pH 5.2 and shaken for 24 h at room temperature, then the membrane with immobilized urease was washed quickly with distilled water and transferred to a vessel with 10 ml 0.1 phosphate buffer (pH 8.0) and 20 mg neutral red and shaken for 24 more hours at room temperature. The membrane with immobilized indicator and enzyme was washed successively with distilled water and 3 N sodium chloride solution during shaking until the complete elimination of nonbonded indicator. The washed wet membrane was stored in a refrigerator at 4°C. The enzyme activity was determined as described[6]. One unit of enzyme activity was taken to be the release of 1 μmol of ammonia per 1 min at 25°C and pH 7.0. The amount of immobilized protein was determined by a modified Lowry method[7].

The spectrophotometric measurements were made on a Perkin Elmer Lambda 2 spectrophotometer. The membranes with immobilized indicator were stretched vertically inside the cuvette using a special frame as described[3].

A Statgraphics® plus software program processed the curves obtained.

## CONCLUSION

As a result of our studies it may be concluded that the method described here permits fast and simple determination of urea concentrations by measuring the change in absorption as a result of the enzyme reaction. The membranes used for immobilization of urease and indicator are waste material from film membranes. Simultaneous immobilization of urease and neutral red permits repeated measurements, between which it is only necessary to wash the membrane. The method can be recommended for determination of urea in water and soil with the use of both types of membranes in the large range of urea concentrations.

## BIOSKETCH

**Milka Krysteva,** PhD, DSc, is a Professor at the Department of Biotechnology, University of Chemical Technology and Metallurgy, 8 Kl.Ohridski str. Sofia 1756, Bulgaria Tel/Fax 359 2 68 34 60 ; e-mail krysteva@uctm.edu. *Lecture courses* include Applied Enzymology; Biotechnological methods in Ecology; and Chemistry of biologically active compounds. *Research interest* are Enzymes-structure and function; Immobilization of enzymes, Preparation of membranes with immobilized enzymes for biosensors, Application of immobilized enzymes for detection of different compounds, Immobilization of cells and their application for production of different steroids, biogas and hydrogen, and Immobilization of photosensitizers and application for water sterilization.
